# Intake of a cetoleic acid concentrate lowered concentrations of markers of inflammation and macrophage infiltration but did probably not increase EPA biosynthesis in male obese Zucker fa/fa rats

**DOI:** 10.1017/S0007114525105394

**Published:** 2025-12-14

**Authors:** Andrea Hansen, Svein Are Mjøs, Eirik Søfteland, Oddrun A. Gudbrandsen

**Affiliations:** 1 Dietary Protein Research Group, Centre for Nutrition, Department of Clinical Medicine, University of Bergen, Bergen 5021, Norway; 2 Department of Chemistry, University of Bergen, 5020 Bergen, Norway; 3 Department of Medicine, Haukeland University Hospital, Bergen, Norway

**Keywords:** Cetoleic acid, *n*-11 MUFA, Herring oil, Inflammation, EPA

## Abstract

Obesity is characterised by chronic low-grade inflammation, which is a key factor in the development of obesity-related co-morbidities. Intake of *n*-3 long-chain PUFAs is associated with anti-inflammatory effects. Recent studies suggest that also *n*-11 long-chain MUFAs may reduce the concentrations of inflammatory markers, possibly by increasing the biosynthesis of EPA. The primary aim was to investigate if diets added herring oil containing cetoleic acid (CA, C22:1*n*-11) or a CA concentrate (CECO) affected the fatty acid composition in tissues from obese rats with chronic inflammation. Secondary aims included investigating the effects on inflammatory markers. Thirty male obese Zucker fa/fa rats were fed diets containing herring oil (HERO) or a CECO, containing 0·70 or 1·40 wt% CA, respectively, with a comparable content of EPA (0·17 and 0·20 wt%, respectively), or a control diet with soyabean oil for 5 weeks. Data were analysed using one-way ANOVA. CA from HERO and CECO diets were recovered in liver, adipose tissue, muscle and blood cells. The EPA concentration was similar between HERO and CECO groups in tissues, whereas the hepatic concentrations of fatty acid desaturases were lower or similar to Controls. The concentrations of TNF*α*, matrix metalloproteinase-3, IL6, monocyte chemotactic protein 1 and integrin *α* M in adipose tissue, and the hepatic concentration of CD68 were lower after CECO intake but were not affected by the HERO diet. To conclude, rats fed the CECO diet had lower concentrations of inflammatory and macrophage infiltration markers, but this effect was probably not mediated through increased EPA biosynthesis.

The WHO has estimated that in 2022, 890 million adults were living with obesity, corresponding to 16 % of all adults over 18 years of age^(1)^. Even more worrying is the approximation that 160 million children and adolescents had obesity in 2022^(1)^. Obesity is a complex disease and is often characterised by chronic low-grade inflammation, which has been suggested to be a key factor in the development of obesity-related co-morbidities including insulin resistance and CVD^(2–4)^.

Fish intake and supplementation with fish oils or *n*-3 PUFA supplements have been shown to be independently associated with lower concentrations of circulating inflammatory markers in several studies, thus indicating inverse relationship between fish consumption and low-grade inflammation^(5–9)^. The anti-inflammatory effect of fish intake is often ascribed to the marine *n*-3 PUFAs EPA and DHA, which may have direct effects as precursors for anti-inflammatory compounds such as prostaglandins, leukotrienes and resolvins, or by replacing arachidonic acid (AA) which is a precursor for pro-inflammatory eicosanoids^(10)^. MUFAs have also been suggested to have anti-inflammatory effects, and fish oils and MUFA concentrates with high cetoleic acid (CA, C22:1*n*-11) and gondoic acid (C20:1*n*-9) contents have been shown to have a lowering effect on inflammatory markers in adipose tissues, abdominal aorta and serum from mice^(11–15)^. A proposed mechanism behind the anti-inflammatory effect of LC-MUFAs such as CA, based on findings in cell culture studies, is that CA stimulates endogenous synthesis of EPA from *α*-linolenic acid by upregulating fatty acid desaturases 1 and 2 (FADS1 and FADS2) mRNAs in the liver^(16)^.

We have recently presented findings that indicate that herring oil, which has a relatively high content of CA, did not increase the protein contents of FADS1 and FADS2 in liver and skeletal muscle of Zucker Diabetic Sprague Dawley (ZDSD) rats^(17)^. Furthermore, based on tissue fatty acid compositions, we found no signs that herring oil increased the endogenous synthesis of EPA and DHA in liver, skeletal muscle, white adipose tissue, blood cells or brain when compared with rats fed a fish oil diet with a comparable content of EPA and DHA but devoid of CA^(17)^. These observations of the effects of fish oil intake on desaturases may be specific to rats with overt type 2 diabetes such as the ZDSD rats, since diabetes and obesity influence the gene expressions and activities of the desaturases in liver of rats^(18,19)^. It is therefore of outermost interest to map in which tissues CA is incorporated in normoglycemic rats and whether the fatty acid compositions in various tissues are affected, and to explore if diets containing CA could affect the tissue concentrations of inflammatory markers.

The obese Zucker fa/fa rat is a much used model for studies on metabolic complications and possible treatments of obesity, since this rat presents a range of abnormalities similar to those seen in humans with obesity^(20)^. This rat becomes obese before the age of 5 weeks and spontaneously develops hyperlipidaemia, hypertension and fatty liver whilst being normoglycemic^(21,22)^. The obesity develops due to a defect in the leptin receptor^(23)^ resulting in hyperphagia^(21)^. The obese Zucker fa/fa rat also has a higher efficiency for food utilisation, a higher rate of lipogenesis in adipose tissues and in the liver^(21,24)^, and a lower capacity for fatty acid oxidation in the liver^(24)^ compared with lean rats. The obese Zucker fa/fa rat experiences a chronic inflammation, with abnormally high concentrations of pro-inflammatory cytokines^(25–28)^.

The main aim of the present study was to investigate how diets containing herring oil or a CA concentrate affected the fatty acid composition in tissues from obese Zucker fa/fa rats. Our hypothesis was that the effects of herring oil and a CA concentrate on the fatty acid compositions in tissues would reflect the fatty acid composition in the diets, with no additional increase in the EPA concentration in response to any up-regulation of FADS2 and FADS1. The secondary aims were to investigate any effects on the concentrations of inflammatory markers in liver and adipose tissues and to examine if differences in fatty acid composition is related to the concentrations of inflammatory markers. To accomplish this, the fatty acid compositions in liver, white adipose tissue, blood cells, skeletal muscle and brain were analysed in obese Zucker fa/fa rats with chronic inflammation after consuming diets added herring oil or a CA concentrate, containing 0·70 or 1·40 wt% CA, respectively, with a comparable content of EPA (0·17 and 0·20 wt%, respectively). A selection of markers of inflammation and macrophage infiltration was measured in white adipose tissue and liver.

## Methods

### Animals and diets

Thirty male obese Zucker fa/fa rats (HsdHlr:ZUCKER-Lepr^fa^) were obtained from Harlan Laboratories (Indianapolis), aged 4–6 weeks at arrival and aged 12 weeks at euthanisation. The rats were housed in pairs in individually ventilated cages (IVC-4, 1500U Eurostandard Type IV S cages, Blue Line, Tecniplast) with free access to chow (Teklad Global Diet 2018 S, Inotiv) until they reached 7 weeks of age. The rats were randomly assigned to receive one of the three experimental diets by drawing paper lots from a jar, each group consisting of ten rats. The rats were given random numbers that could not be linked to the experimental group. The experimental semi-purified diets were modified versions of the American Institute of Nutrition’s recommendation for growing laboratory rodents (AIN-93G)^(29)^ with the addition of 1·6 g methionine/kg diet as recommended by Reeves^(30)^ and differed only in their lipid sources (Table 1). All diets contained 20wt% protein (casein), 10wt% fat and 11·8wt% sucrose and had comparable energy content. The two intervention diets contained either refined oil prepared from herring (*Clupea harengus*) residuals (the HERO diet) or a CA concentrate produced from refined herring oil extracted from herring trimmings (CECO diet), designed with twice as high CA content in the CECO diet compared with the HERO diet. The fatty acids in the herring oil were transesterified to ethyl esters and distilled to separate the fatty acids, and the ethyl esters were enzymatically re-esterified to triacylglycerols using glycerol with a maximum of 5 % ethyl esters in the final product. The production process included multiple purification steps to remove unwanted compounds, such as environmental contaminants. All diets contained adequate amounts of essential fatty acids according to Reeves et al.^(29)^. The control diet contained soyabean oil as the only lipid source. All ingredients were purchased from Dyets Inc. except casein which was purchased from Sigma-Aldrich, herring oil from Pelagia AS and the CA concentrate from Epax Norway AS. The rats had *ad libitum* access to feed and water. The diets were stored at –15°C, and daily portions were thawed in the morning.

**Table 1.** Compositions of the experimental diets

Contents (g/100 g diet)	Control diet	HERO diet	CECO diet
Soyabean oil	10·00	6·30	5·75
Herring oil	–	3·70	–
CECO	–	–	4·25
Casein^*^	21·56	21·56	21·56
Sucrose	9·00	9·00	9·00
Cornstarch	48·23	48·23	48·23
Cellulose	5·00	5·00	5·00
t-Butylhydroquinone	0·0014	0·0014	0·0014
Mineral mix (AIN-93-MX)^†^	3·50	3·50	3·50
Vitamin mix (AIN-93-VX)^‡^	1·00	1·00	1·00
Growth and maintenance supplement^§^	1·00	1·00	1·00
l-methionine	0·16	0·16	0·16
l-cystine	0·30	0·30	0·30
Choline bitartrate^**^	0·25	0·25	0·25

HERO, herring oil; CECO, cetoleic acid concentrate.

* Contains 91·9 % crude protein (high-fat diet) or 92·78 % crude protein (other diets).

† Contains sucrose (221 g/kg).

‡ Contains sucrose (967 g/kg).

§ Contains vitamin B_12_ (40 mg/kg) and vitamin K_1_ (25 mg/kg) mixed with sucrose (995 g/kg) and dextrose (5 g/kg).

** Contains 41 % choline.

The rats were gradually habituated to semi-purified powder diets (AIN-93G) for growing rodents^(29)^ for 4 d before they were moved to large open cages (W115xD67·5xH153 cm Suite Royale XL, Savic®). Three large open cages (one per experimental group) each consisted of two plastic floors and four plastic platforms with metal ladders. Each cage was split in two equal-size halves, with 2HK Nestpak (TAPVEI ® Harjumaa, Estonia OÜ) on plastic floors and platforms, housing six rats on the upper half and four rats on the lower half of the cage. Both halves of the cage were equipped with the following environmental enrichments: three Fat Rat Huts (red polycarbonate, size 150 mm × 165 mm × 85 mm, Datesand), two cardboard tunnels (Play Tunnel, 100 × 50·8 × 1·25 mm, Datesand), two gnawing blocks (Aspen brick, 100 mm × 20 mm × 20 mm, TAPVEI ®), one Rat Corner Home (paper pulp, 23 × 19 × 9·5 cm, Datesand), two 8 g portions of Bed-R’Nest nesting material (kraft paper, The Andersons, Inc.), one wooden house (pine, 28 × 16 × 18 cm, Trixie Heimtierbedarf GmbH & Co), one wooden playing roll with a bell (Trixie), one suspension bridge (wooden ladder with rope ladder, play rope and rope ring with wooden block, Trixie), one-two plastic bottles with water (Classic Crystal Deluxe Water Drinking Bottle, Caldex Holdings Ltd.) and ceramic bowls containing water or powder feed. The rats were housed in a room with controlled light/dark cycle (dark 20.00–06.00) and temperature 22–23°C.

### Design

The rats were weighed three times per week. After 23 d of feeding the experimental powder diets, the rats were housed individually in metabolic cages (Ancare Corp.) for 24 h for measurement of feed intake, without fasting in advance. At the end of the experimental period, that is, after 35 d with powder feed, the feed was withdrawn between 06.00 and 07.00, and rats were fasted for 6 h with free access to drinking water before being euthanised while anaesthetised with isoflurane (Isoba vet, Intervet, Schering-Plough Animal Health) mixed with oxygen. The body length was measured with a ruler while the rats were anaesthetised. Blood was drawn from the heart and collected in BD Vacutainer SST II Advance gel tubes (Becton, Dickinson and Company) for isolation of serum, and in BD Vacutainer K2EDTA tubes (Becton, Dickinson and Company) for separation of blood cells from plasma. The vacutainers were centrifuged (2000 × g), and serum and blood cells were frozen at –80°C. The liver, the left kidney and epididymal white adipose tissues (WATepi) from both sides were carefully dissected out and weighed. The skeletal muscle from the thigh and brain were dissected out. All tissues were frozen at –80°C until analysed.

The personnel handling the rats and conducting the analyses were blinded to the rats’ group allocation. The rats were handled and euthanised in random order.

### Determination of fatty acid compositions in diets and tissues

Lipids were extracted from diets, liver and thigh muscle by the method of Bligh and Dyer^(31)^, added to heneicosanoic acid (C21:0) as internal standard and were methylated as previously described^(32)^. Samples of blood cells, WATepi and brain were added to heneicosanoic acid as internal standard and were methylated without prior extraction of lipids as described previously^(32)^. The methyl ester samples were quantified using an Agilent 7890 gas chromatograph equipped with a flame ionisation detector (Agilent Technologies, Inc.) and a BPX-70 capillary column (SGE Analytical Science) as described in Sciotto & Mjøs^(33)^ with minor adjustments of the temperature programme to provide baseline resolution between *n*-9 and *n*-11 monoenoic isomers. To assure accurate quantitative amounts, chromatographic areas were adjusted with empirical response factors based on the GLC-793 reference mixture (Nu-Chek Prep). The samples were run in randomised order, and a reference mixture was run as every eighth sample (or more often) in the chromatographic sequences. The compounds were identified by GC-MS using the methodology described in Wasta & Mjøs^(34)^.

### Quantification of TAG in liver

Lipids were extracted from the liver according to the method by Bligh and Dyer^(31)^, evaporated to dryness under nitrogen and re-dissolved in isopropanol before quantification of TAG on the Cobas c111 system using the TRIGL (Triglycerides) kit from Roche Diagnostics.

### Energetic contents in the diets

The dietary energetic content was measured by Nofima BioLab by a bomb calorimeter method in accordance with ISO9831:1998^(35)^.

### Protein analyses in liver, muscle and adipose tissue

Samples of liver, WATepi and thigh muscle were homogenised in PBS. The tissue protein contents were measured using the Bradford dye-binding method^(36)^, with protein assay dye reagent (Bio-Rad Laboratories) and bovine serum albumin (Bio-Rad Protein Assay Standard II, Bio-Rad Laboratories) as the standard. FADS2 (also known as delta-6 desaturase) was measured using the Rat Delta-6 Desaturase/FADS2 ELISA Kit (Sandwich ELISA) cat no LS-F7004 (LifeSpan BioSciences, Inc.). FADS1 (also known as delta-5 desaturase) was measured using the Rat FADS1 ELISA Kit (Sandwich ELISA) cat no LS-F56186 (LifeSpan BioSciences, Inc.). Fatty acid elongase-2 (Elovl2) was measured using the Rat Elovl2 (elongation of very long-chain fatty acids protein 2) ELISA Kit (sandwich ELISA), cat no EKX-K4OWMF (Nordic Biosite). Stearoyl-CoA desaturase 1 (SCD1, also known as delta-9 desaturase) was measured using the Rat SCD1/SCD ELISA Kit (Sandwich ELISA) cat no LS-F32611 (LifeSpan BioSciences, Inc.). TNF*α* was measured using the Rat TNF Alpha ELISA Kit (Sandwich ELISA) cat no LS-F5193 (LifeSpan BioSciences, Inc.). Matrix metalloproteinase-3 (MMP3) was measured using the Rat MMP3 ELISA Kit (Sandwich ELISA) cat no LS-F32505 (LifeSpan BioSciences, Inc.). IL6 was measured using the Rat IL6/IL6 Kit (Sandwich ELISA) cat no LS-F9753 (LifeSpan BioSciences, Inc.). Monocyte chemotactic protein 1 (MCP1, also known as C-C motif chemokine 2) was measured using the Rat CCL2/MCP1 Kit (Sandwich ELISA) ELISA cat no F37066 (LifeSpan BioSciences, Inc.). Integrin *α* M (ITGAM, also known as cluster of differentiation molecule 11B (CD11B)) was measured using the Rat ITGAM/CD11b ELISA Kit (Sandwich ELISA) cat no LS-F4904 (LifeSpan BioSciences, Inc.). Cluster of Differentiation 68 (CD68) was measured using the Rat CD68 ELISA Kit (Sandwich ELISA) cat no LS-F33168 (LifeSpan BioSciences, Inc.). Plates were read at SpectraMax Plus384 Microplate Reader (Molecular Devices) or Multiscan FC (Thermo Scientific), and all samples were analysed simultaneously in the same plate from each of the ELISA assays, with CV < 5 %. Concentrations are presented relative to total protein content.

### Biochemical analyses in serum and urine

Concentrations of alanine transaminase and aspartate transaminase in serum were measured on the Cobas c111 system (Roche Diagnostics) using the ALTL (alanine aminotransferase acc. IFCC, measured with pyridoxal phosphate activation) and ASTL (aspartate aminotransferase acc. IFCC, measured with pyridoxal phosphate activation) kits from Roche Diagnostics. Serum N-terminal Prohormone Brain Natriuretic Peptide (NT-proBNP) was quantified using the Rat NT-proBNP (Sandwich ELISA) ELISA Kit (cat. no. LS-F23593) from LifeSpan BioSciences, Inc.

Urine concentrations of T cell immunoglobulin mucin-1 (TIM-1) and cystatin C were quantified using the Rat TIM-1/KIM-1/HAVCR Quantikine ELISA kit (cat. no. RKM100) and the Mouse/Rat Cystatin C Quantikine ELISA (cat. no. MSCTC0) from R&D Systems, Bio-Techne. Creatinine in urine was quantified using the CREP2 (creatinine plus ver.2) kit from Roche Diagnostics.

Plates with NT-proBNP, Cystatin C and TIM-1 were read on a SpectraMax Plus384 Microplate Reader. All samples were analysed simultaneously in the same plate from each of the ELISA assays, with CV < 5 %.

### Testing of glucose tolerance

To ensure that the rats included in the statistical analyses were non-diabetic, all rats underwent a meal glucose tolerance test at day 28. Rats were housed individually while fasted for 10 h (from 06.30), and glucose was monitored in fasting state and 120 min after receiving an oral dose of 2 g dextrose + sucrose per kg BW. The dextrose + sucrose load was prepared as a modified, semi-purified meal on the basis of AIN-93G^(29)^ with 417 g of dextrose + sucrose per kg diet (composition of the diet for meal tolerance test: 70 g soyabean oil, 262 g casein (76 % raw protein; Sigma-Aldrich), 390 g dextrose, 165 g cornstarch, 50 g cellulose, 35 g mineral mix (containing 22·1 % sucrose), 10 g vitamin mix (containing 96·7 % sucrose), 3 g L-cystine, 1·6 g L-methionine, 2·5 g choline bitartrate and 10 g growth and maintenance supplement (containing 99·5 % sucrose) per kilo diet; all ingredients except casein were purchased from Dyets Inc). The rats were allowed a maximum of 15 min to finish the meal. The dorsal tail vein was punctuated, and blood glucose was measured using a blood glucose measuring device (Contour next; Bayer Consumer Care AG). In addition, glucose/creatinine ratio was measured in urine collected on day 23, using the GLUC2 (glucose HK) and CREP2 kits from Roche Diagnostics.

### Outcome measurements

The primary outcome was to investigate how intake of herring oil and a CA concentrate would affect fatty acid composition in tissues from obese Zucker fa/fa rats. The secondary outcomes were to investigate if consumption of herring oil or CA concentrate affected the concentrations of markers of inflammation and macrophage infiltration in liver and WATepi, and if this was associated with fatty acid compositions in these tissues.

### Statistical analyses

This is the first study to investigate the effects of herring oil and a CA-rich concentrate on fatty acid composition in tissues and inflammatory responses in obese Zucker fa/fa rats. Therefore, no data on effect size were available for sample size calculation or minimally detectable effect sizes. Based on studies conducted in rats and mice using CA-rich fish oils with group sizes of six to twelve rodents/group^(37)^, and our recent study using ZDSD rats fed diets containing herring oil, anchovy oil or soyabean oil, we designed this study with ten rats per experimental group. One rat in the HERO group developed diabetes and was therefore excluded from statistical analyses (described in more detail under the Results section); therefore, statistical analyses were conducted with ten rats in the Control group, nine rats in the HERO group and ten rats in the CECO group.

Statistical analyses were conducted using SPSS Statistics version 29 (SPSS, Inc., IBM Company). All data were evaluated for normality using the Shapiro–Wilks test, revealing that most variables were normally distributed; therefore, one-way ANOVA was used to compare the experimental groups. Since this study is regarded as an exploratory study without the possibility of a proper calculation of the necessary sample size, when appropriate, the ANOVA analyses were followed by Tukey’s HSD *post hoc* test as recommended by Lee et al.^(38)^. The cut-off value for statistical significance was set at a probability of 0·05.

## Results

### Description of the diets

The experimental diets were similar with regard to all ingredients except the sources of fat (Table 1) and had similar energy content of around 19 kJ/g diet. The diets were added 10 wt% fat, either as solely soyabean oil in the Control diet, a combination of 3·70 wt% herring oil and 6·30 wt% soyabean oil in the HERO diet, or a combination of 4·25 wt% CA concentrate and 5·75 wt% soyabean oil in the CECO diet. The diets were designed to contain twice as much CA in the CECO diet compared with the HERO diet (1·40 *v*. 0·70 wt%, respectively, Table 2). Gadoleic acid (GA, C20:1*n*-11) and 7-octadecenoic acid (7OA, C18:1*n*-11) were found in the HERO diet, and GA but not 7OA was found in the CECO diet. The Control diet did not contain CA, GA or 7OA. *α*-Linolenic acid (C18:3*n*-3) was found in all three diets, whereas the longer *n*-3 PUFAs stearidonic acid (C18:4*n*-3), EPA, docosapentaenoic acid (C22:5*n*-3) and DHA were found in the HERO diet and in the CECO diet but not in the Control diet. The amount of EPA was comparable between the HERO and the CECO diets (0·17 and 0·20 wt%, respectively), whereas the DHA content was markedly higher in the HERO diet compared with the CECO diet (0·18 and 0·04 wt%, respectively). Linoleic acid (C18:2*n*-6) and oleic acid (C18:1*n*-9) mainly originated from the soyabean oil added to the diets and were found in all three diets. The CECO diet had a higher content of gondoic acid (C20:1*n*-9) compared with the HERO diet (0·75 and 0·35 wt%, respectively), whereas the Control diet contained a very low amount of gondoic acid. Small amounts of erucic acid (C22:1*n*-9) and nervonic acid (C24:1*n*-9) were found only in the HERO diet and the CECO diet.

**Table 2.** Contents of fatty acids in the diets

g/100 g diet	Control diet	HERO diet	CECO diet
C14:0	0·02	0·26	0·06
C15:0	<LOQ	0·02	<LOQ
C16:0	0·92	0·95	0·58
C17:0	0·01	0·01	0·01
C18:0	0·31	0·24	0·21
C20:0	0·02	0·02	0·02
C22:0	0·02	0·02	0·02
C16:1 *n*-7	0·01	0·13	0·07
C18:1 *n*-5	<LOQ	0·01	0·01
C18:1 *n*-7	0·12	0·11	0·10
C18:1 *n*-9	1·71	1·31	1·17
C20:1 *n*-9	0·01	0·35	0·75
C22:1 *n*-9	<LOQ	0·03	0·08
C24:1 *n*-9	<LOQ	0·03	0·05
C18:1 *n*-11	<LOQ	0·04	<LOQ
C20:1 *n*-11	<LOQ	0·05	0·08
C22:1 *n*-11	<LOQ	0·70	1·40
C16:2 *n*-4	<LOQ	0·02	0·01
C16:3 *n*-4	<LOQ	0·01	0·01
C18:2 *n*-6	4·52	2·92	2·58
C18:3 *n*-3	0·57	0·39	0·34
C18:4 *n*-3	<LOQ	0·07	0·03
C20:5 *n*-3	<LOQ	0·17	0·20
C22:5 *n*-3	<LOQ	0·02	0·01
C22:6 *n*-3	<LOQ	0·18	0·04

HERO, herring oil; CECO, cetoleic acid concentrate; LOQ, level of quantification.

The following fatty acids were below LOQ in all diets: C12:0, C23:0, C24:0, C14:1 *n*-5, C16:1 *n*-9, C17:1*n*-7, C17:1 *n*-8, C18:1*n*-9t, C20:1*n*-7, C22:1*n*-7, C18:2*n*-6tc, C18:3*n*-6, C18:2 *n*-4, C20:2*n*-6, C20:3*n*-6, C20:4*n*-6, C20:3*n*-3, C20:4 *n*-3, C21:5 *n*-3, C22:4*n*-6 and C22:5*n*-6.

### Description of the rats

One rat in the HERO group was excluded from the study based on the results from the meal glucose tolerance test and the measurement of the urine glucose/creatinine ratio showing that this rat had poor glucose tolerance and was developing type 2 diabetes. The serum concentrations of alanine transaminase, aspartate transaminase and NT-proBNP (markers of liver and heart injury) were similar between the groups (Table 3). The urine concentrations (relative to creatinine) of markers of kidney damage, that is, cystatin C and TIM-1, were similar between the groups (Table 3).

**Table 3.** Safety biomarkers measured in serum or urine at endpoint (day 35)

	Control group		HERO group		CECO group		*P* ANOVA
	Mean	sd	Mean	sd	Mean	sd	
Serum alanine transaminase (U/L)	89·9	21·7	91·1	26·5	78·2	22·6	0·42
Serum aspartate transaminase (U/L)	144·0	40·0	142·2	40·7	114·6	26·9	0·15
Serum NT-proBNP (ng/ml)	12·3	2·5	14·9	2·9	13·6	4·3	0·21
Urine cystatin C (µg/mmol creatinine)	1356	687	1109	267	951	273	0·16
Urine TIM-1 (ng/mmol creatinine)	246	138	215	37	209	32	0·59

HERO, herring oil; CECO, cetoleic acid concentrate; TIM-1, T cell immunoglobulin mucin-1; NT-proBNP, N-terminal Prohormone Brain Natriuretic Peptide.

Data are presented as mean (SD) for *n* 10 in the Control group, *n* 9 in the HERO group and *n* 10 in the CECO group. Groups are compared using one-way ANOVA, and *P* < 0·05 was considered significant.

The feed intake was similar between the groups (Table 4). The groups were similar with regard to body weight at baseline and at endpoint, with no differences between the groups for body weight gain, body weight to square body length ratio, hepatic TAG content, or relative weights of liver, the left kidney and WATepi from both sides (Table 4).

**Table 4.** Feed intake registered during 24-h housing in metabolic cage, body weight at baseline and endpoint (day 35), change in body weight, body weight to square body length ratio, hepatic TAG content, and relative weights of the liver, the left kidney and epididymal white adipose tissue from both sides

	Control group		HERO group		CECO group		*P* ANOVA
	Mean	sd	Mean	sd	Mean	sd	
Feed intake (kJ/kgBW/24 h)	831	142	903	129	905	237	0·58
Body weight (g) at baseline	303	9	299	8	302	12	0·72
Body weight (g) at endpoint	529	31	528	28	531	32	0·98
Body weight gain (%)	75	11	77	11	76	13	0·93
Body weight to square body length ratio (kg/m^2^) at endpoint	10·0	0·5	9·8	0·4	9·9	0·4	0·60
Liver TAG content (µmol/g) at endpoint	251	41	264	90	267	63	0·85
Relative liver weight (g/kg body weight) at endpoint	43·7	6·1	45·9	7·4	46·0	4·8	0·66
Relative weight of left kidney (g/kg body weight) at endpoint	2·35	0·19	2·36	0·18	2·34	0·21	0·98
Relative WATepi weight (g/kg body weight) at endpoint	27·4	3·6	27·9	2·5	28·1	2·8	0·88

HERO, herring oil; CECO, cetoleic acid concentrate.

Data are presented as mean (SD) for *n* 10 in the Control group, *n* 9 in the HERO group and *n* 10 in the CECO group. Groups are compared using one-way ANOVA, and *P* < 0·05 was considered significant.

### Fatty acids and desaturases

**Figure 1.**
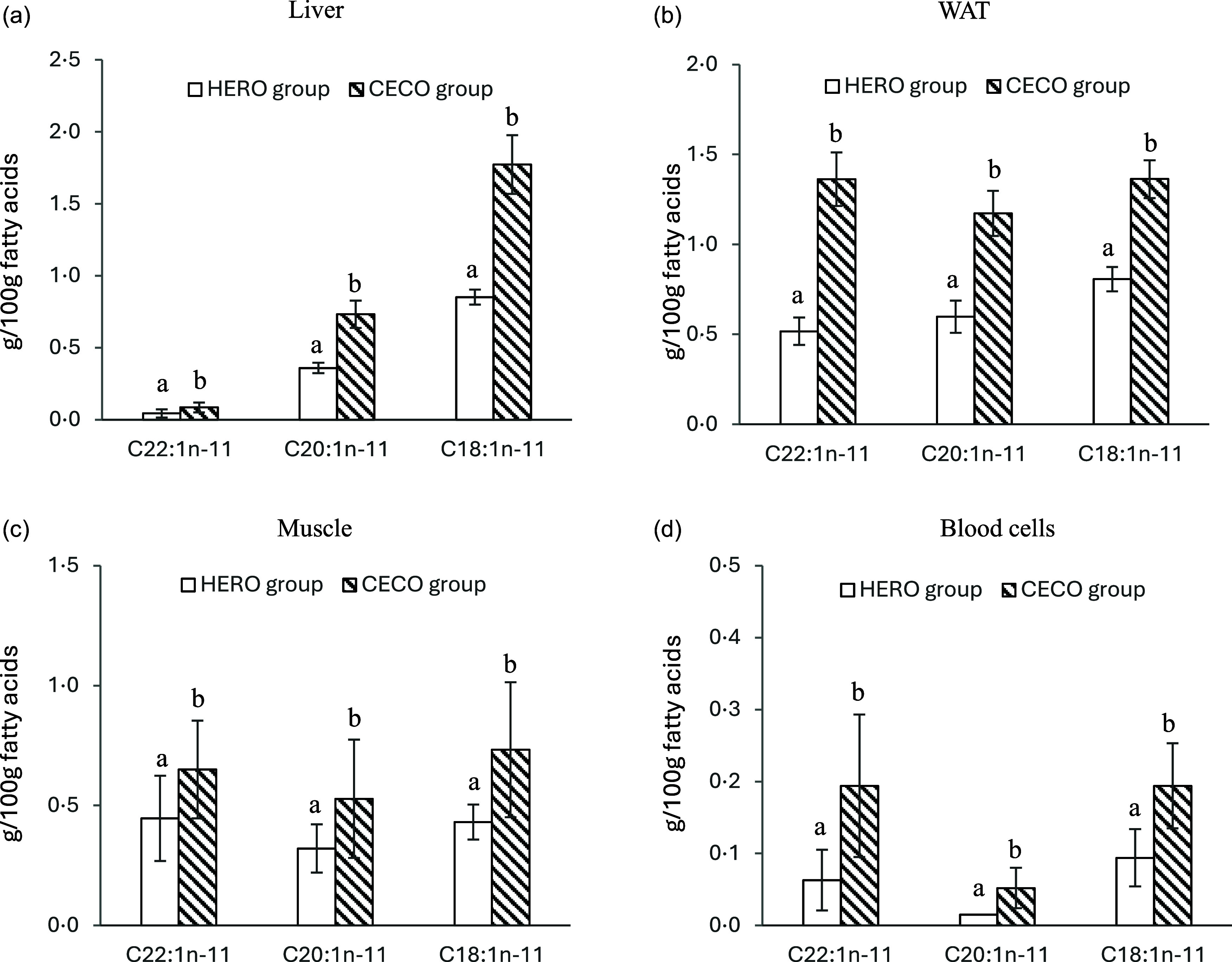
Relative weights of *n*-11 MUFA in liver (a), epididymal white adipose tissue (WAT) (b), skeletal muscle (muscle) (c) and blood cells (BC) (d). *n*-11 MUFAs were not recovered in rats fed the Control diet. Data are presented as mean and standard deviation for *n* 10 in the Control group, *n* 9 in the HERO group, and *n* 10 in the CECO group. Groups are compared using Student’s *t* test. Bars with different letters are significantly different (*P* < 0·05). CA, GA and 7OH was not detected in brain. HERO, herring oil; CECO, cetoleic acid concentrate.

CA and its two shorter metabolites GA and 7OA were found in liver, blood cells, skeletal muscle and WATepi in rats in the HERO and CECO groups (Figure 1 and online Supplementary Tables 1–5), whereas no *n*-11 MUFAs were found in the brain in any of the rats in these groups. CA, GA and 7OA were not identified in any tissues from the Control rats. The relative amounts (wt%) of CA, GA and 7OA were higher in liver, blood cells, muscle and WAT from rats in the CECO group compared with the HERO group. Gondoic acid was recovered in liver, blood cells, muscle and WATepi; CECO fed rats had the highest and the Control rats had the lowest amount of gondoic acid in these tissues. Gondoic acid was not found in the brain in any of the rats in the experimental groups.

**Figure 2.**
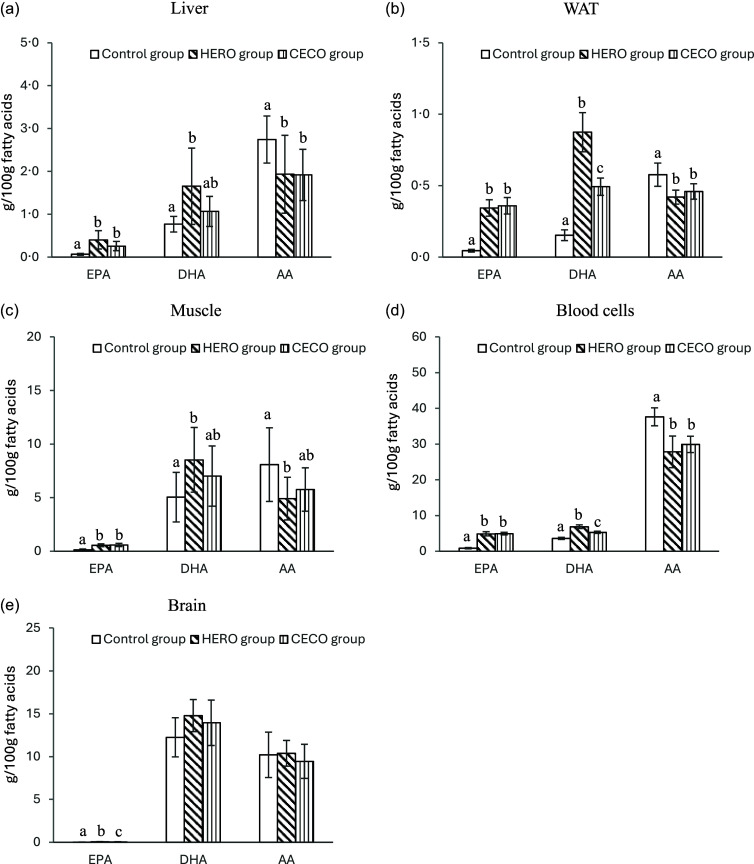
Relative weights (g/100 g FA) of EPA, DHA and AA in liver (a), epididymal white adipose tissue (WAT) (b), skeletal muscle (muscle) (c), blood cells (BC) (d) and brain (e). Data are presented as mean and standard deviation for *n* 10 in the Control group, *n* 9 in the HERO group and *n* 10 in the CECO group. Groups are compared using one-way ANOVA followed by Tukey’s HSD *post hoc* test when appropriate. Bars with different letters are significantly different (*P* < 0·05). HERO, herring oil; CECO, cetoleic acid concentrate.

The EPA content was higher in liver, WATepi, blood cells and muscle from rats fed the HERO diet or the CECO diet, with no difference between the two groups, when compared with the Control group (Figure 2 and online Supplementary Tables 1–5). The content of EPA was higher in the brain from rats in the HERO group compared with those in the CECO group, and in both groups the EPA content was higher than in the Control group.

The amount of DHA in liver and muscle were similar between the HERO group and the CECO group (Figure 2 and online Supplementary Tables 1–5). The DHA content was higher in the HERO group compared with the Control group in liver and muscle, with no difference between CECO and Control groups. The brain DHA content was similar in all experimental groups. The DHA content in blood cells and WATepi was higher in the HERO group compared with the CECO group, and both groups had a higher DHA content in blood cells and WATepi compared with the Control group.

The AA (C20:4*n*-6) content in liver, blood cells and WATepi were similar between the HERO and the CECO groups, and levels were lower compared with the Control group (Figure 2 and online Supplementary Tables 1–5). In muscle, the AA content was lower in the HERO group compared with the Control group, with no difference between the CECO group and the Control group, or between the HERO group and the CECO group. The AA content in brain was similar between all three experimental groups.

**Figure 3.**
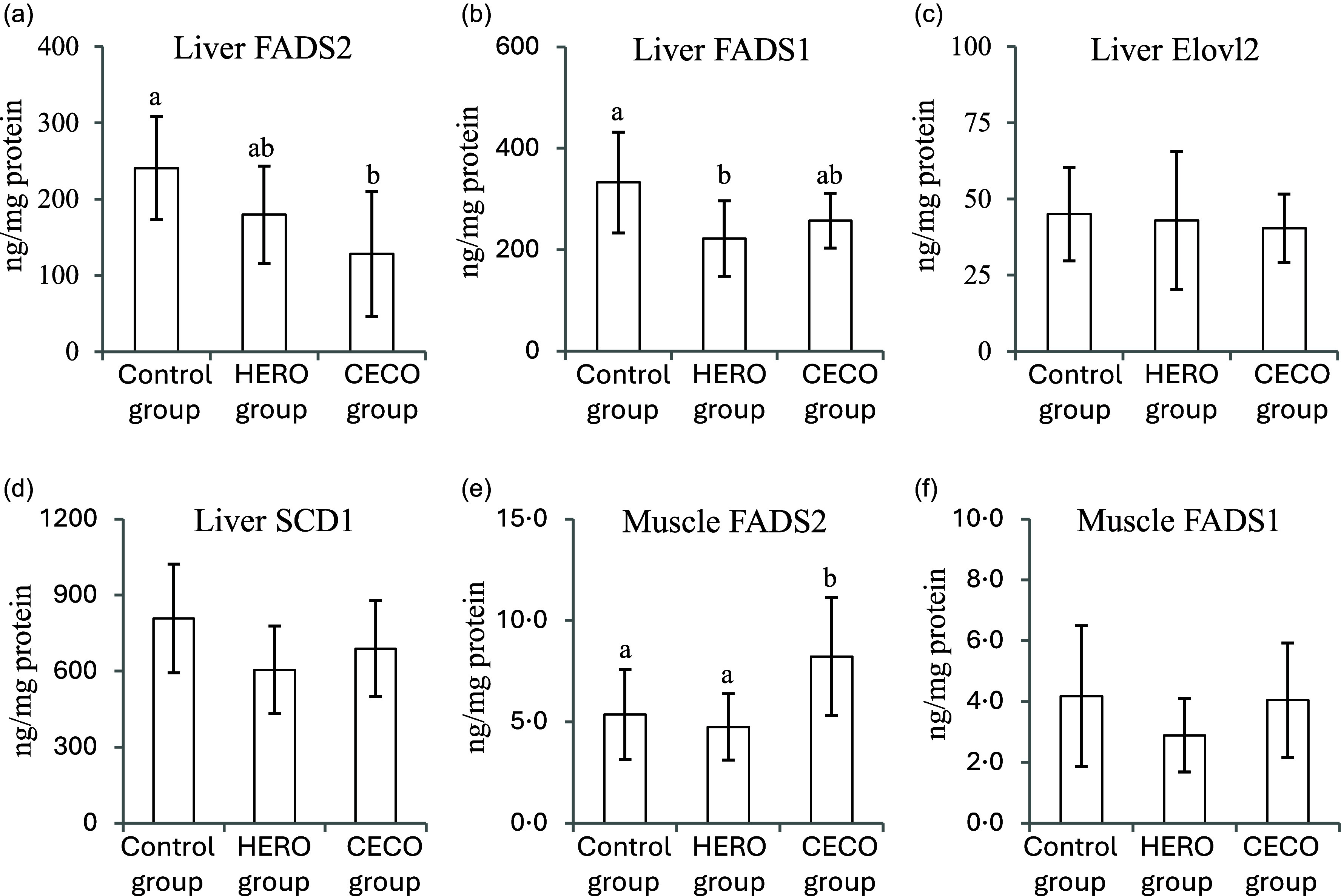
Concentrations (presented relative to protein content) of fatty acid desaturase 2 (FADS2) (a), fatty acid desaturase 1 (FADS1) (b), fatty acid elongase-2 (Elovl2) (c) and stearoyl-CoA desaturase 1 (SCD1) (d) in liver, and of fatty acid desaturase 2 (FADS2) (e) and fatty acid desaturase 1 (FADS1) (f) in muscle. Data are presented as mean and standard deviation *n* 10 in the Control group, *n* 9 in the HERO group and *n* 10 in the CECO group. Groups are compared using one-way ANOVA followed by Tukey’s HSD *post hoc* test when appropriate. Bars with different letters are significantly different (*P* < 0·05). HERO, herring oil; CECO, cetoleic acid concentrate.

The liver concentration of FADS2, catalysing the delta-6 desaturation of C18:2*n*-6 to C18:3*n*-6 and of C18:3*n*-3 to C18:4*n*-3, was lower in the CECO group compared with the Control group but was not affected by the HERO diet (Figure 3(a)). The hepatic FADS1 concentration, catalysing the delta-5 desaturation of C20:3*n*-6 to AA (C20:4*n*-6) and of C20:4*n*-3 to EPA (C20:5*n*-3), was lower in the HERO group compared with the Control group, with no difference between the CECO and Control groups (Figure 3(b)). The liver content of Elovl2, catalysing the elongation of PUFAs with 20–24 carbons, was similar between the groups (Figure 3(c)). Also, the liver concentration of SCD1, catalysing the delta-9 desaturation of SFAs, was similar between the groups (Figure 3(d)). The muscle content of FADS2 was higher in the CECO group when compared with the Control group and the HERO group (Figure 3(e)), whereas the muscle content of FADS1 was similar between all three groups (Figure 3(f)).

### Markers of inflammation and macrophage infiltration

**Figure 4.**
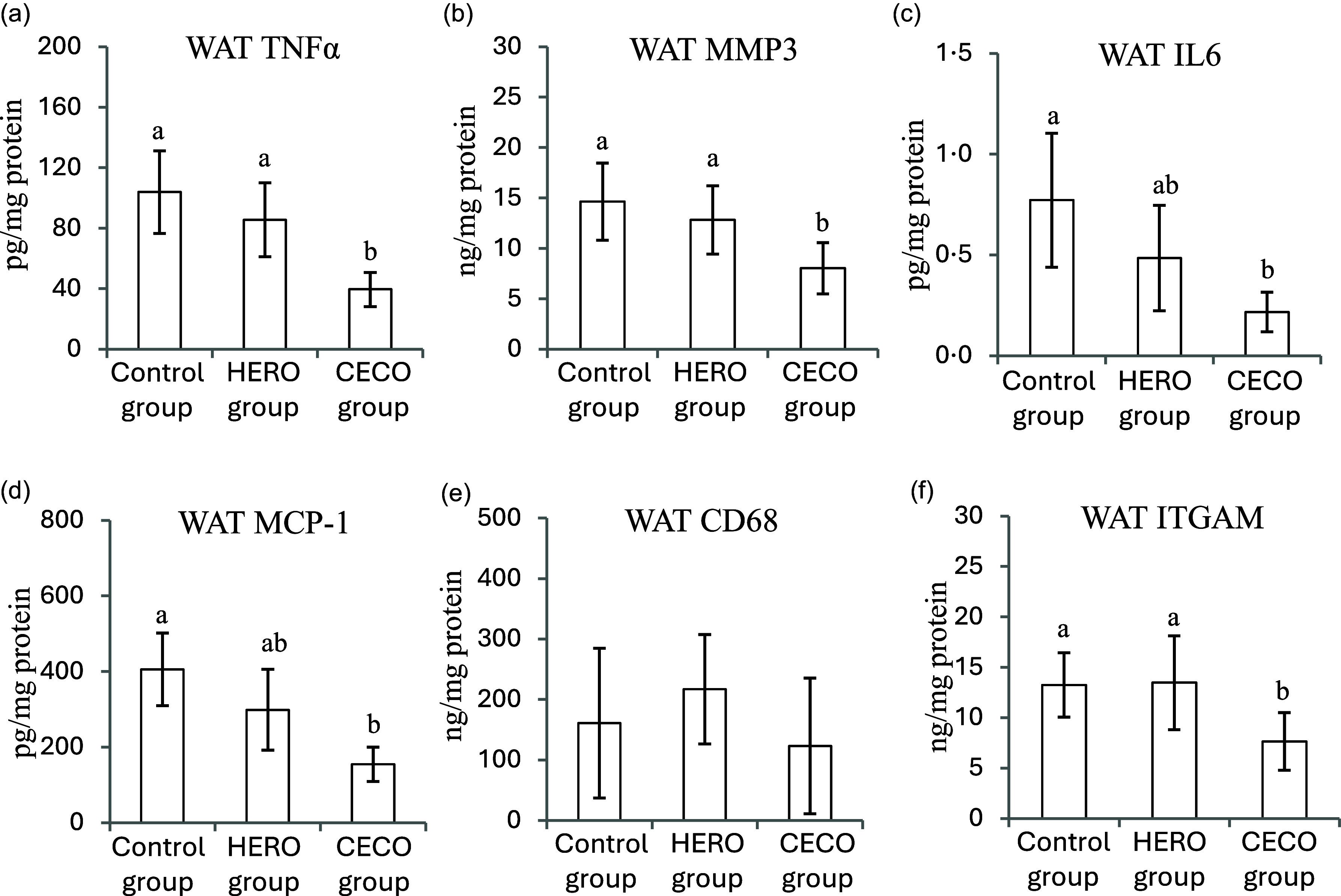
Concentrations (presented relative to total protein content) of tumour necrosis factor *α* (TNF*α*) (a), matrix metalloproteinase-3 (MMP3) (b), IL6 (c), monocyte chemotactic protein 1 (MCP1) (d), cluster of differentiation 68 (CD68) (e) and integrin *α* M (ITGAM) (f) in epididymal white adipose tissue (WAT). Data are presented as mean and standard deviation for *n* 10 in the Control group, *n* 9 in the HERO group and *n* 10 in the CECO group. Groups are compared using one-way ANOVA followed by Tukey’s HSD *post hoc* test when appropriate. Bars with different letters are significantly different (*P* < 0·05). HERO, herring oil; CECO, cetoleic acid concentrate.

The concentrations of the inflammatory markers TNF*α*, MMP3, IL6 and MCP1 were lower in WATepi in the CECO group compared with the Control group, with no difference between the HERO group and the Control group (Figure 4(a)–(d)). The macrophage marker CD68 was similar in WATepi from all groups (Figure 4(e)), whereas the WATepi content of ITGAM, another marker for macrophage infiltration, was lower in the CECO group compared with the HERO group and the Control group, with no difference between the two latter (Figure 4(f)).

In liver, no differences were seen between the experimental groups for concentrations of TNF*α*, MMP3, ITGAM, IL6 or MCP1 (Table 5). The hepatic concentration of CD68 was lower in the CECO group compared with Controls, with no difference between the HERO group and the Control group (Table 5).

**Table 5.** Concentrations (presented relative to total protein content) of markers for inflammation and macrophage infiltration in liver

	Control group		HERO group		CECO group		*P* ANOVA
	Mean	sd	Mean	sd	Mean	sd	
TNF*α* (ng/mg protein)	1·45	0·36	1·40	0·35	1·37	0·27	0·86
MMP3 (ng/mg protein)	624	176	532	250	647	215	0·48
CD68 (ng/mg protein)	2494	673^a^	2003	775^ab^	1480	491^b^	6·9 × 10^–3^
ITGAM (ng/mg protein)	1·97	0·53	2·08	0·62	1·70	0·35	0·25
IL6 (pg/mg protein)	6·68	3·41	8·60	4·37	6·87	1·68	0·40
MCP1 (pg/mg protein)	3920	1388	3492	1997	4041	2537	0·83

HERO, herring oil; CECO, cetoleic acid concentrate; TNF*α*, tumour necrosis factor *α*; MMP3, matrix metalloproteinase-3; CD68, cluster of differentiation 68; ITGAM, integrin *α* M; MCP1, monocyte chemotactic protein 1.

Data are presented as mean (SD) for *n* 10 in the Control group, *n* 9 in the HERO group and *n* 10 in the CECO group. Groups are compared using one-way ANOVA followed by Tukey’s HSD *post hoc* test when appropriate. Means in a row with different letters are significantly different. *P* < 0·05 was considered significant.

## Discussion

In this study, we present evidence that CA, a long-chain *n*-11 MUFA found in oils from pelagic fish such as herring, may lower the concentrations of several inflammatory markers in WATepi, thus indicating an anti-inflammatory effect. It has been speculated that CA induce lowering of the inflammatory status through enhanced endogenous synthesis of EPA; however, this was not supported by our findings. The content of EPA was comparable in the HERO and the CECO diets, whereas the CA content was two times higher in the CECO diet compared with the HERO diet (1·40 *v*. 0·70 wt%, respectively). This was reflected in the tissues from obese Zucker fa/fa rats fed the HERO or the CECO diet, where the concentration of EPA was similar between the HERO and CECO groups in liver, WATepi, blood cells and muscle, and the CA content was around twice as high in tissues from the CECO group compared with the HERO group. Still, the concentrations of several markers of inflammation and macrophage infiltration were lower in WATepi from the CECO group compared with the Control group, whereas the HERO diet had no effect.

We recently presented evidence that CA was metabolised in the ZDSD rat, where it was *β*-oxidised and esterified as phospholipids, triacylglycerols and cholesteryl esters^(17)^. The findings in the obese Zucker fa/fa rats in the present study are in line with our previous paper, with the amounts of CA and its two chain-shortened metabolites in liver, in amounts CA < GA < 7OA in both HERO and CECO groups, indicating that CA was efficiently *β*-oxidised in the liver.

The presence of CA and its shorter metabolites in tissues may affect biochemical pathways including the metabolism of other fatty acids, and it has been proposed that CA stimulates endogenous synthesis of EPA from *α*-linolenic acid by upregulating FADS2 and FADS1 mRNAs in the liver^(16)^. Results in our previous study in ZDSD rats point to no evidence of increased endogenous synthesis of EPA and DHA after CA intake from herring oil^(17)^. The ZDSD rats had overt type 2 diabetes^(17)^, whereas none of the Zucker fa/fa rats included in the present study were diabetic. Both diabetes and obesity influence the gene expressions and the activities of the desaturases in liver of rats^(18,19)^, and we therefore wanted to investigate if the liver and muscle contents of FADS1 and FADS2, as well as tissue contents of EPA, were differently affected by CA intake in ZDSD rats and obese but normoglycemic Zucker fa/fa rats. The higher liver concentration of EPA in the HERO and CECO groups compared with the Control group in the present study is likely a reflection of the EPA content in the diets, since the protein concentrations of FADS2, FADS1 and Elovl2 were not upregulated in groups fed CA compared with Controls. Interestingly, the liver content of FADS2, regarded as the rate-determining enzyme in the LC-PUFA synthesis^(39)^, was almost nine times higher in obese Zucker fa/fa rats compared with the ZDSD rats^(17)^. The FADS2 muscle content was higher in obese Zucker fa/fa rats fed the CECO diet compared with both Control and HERO groups, with no between-group difference for muscle FADS1. Since the FADS2 protein content in muscle was approximately 1/50 of that from liver, the higher FADS2 in muscle from CECO fed rats would probably have little influence on the total EPA production in these rats. In line with our previous findings in the ZDSD experiment, we found no indications that CA stimulated EPA synthesis in the Zucker fa/fa rats.

Adipose tissues play a central role as a source and site of inflammation. Accumulation of immune cells within the adipose tissue may lead to the process underlying the link between obesity and obesity-related morbidities^(40)^, and increased adiposity is often associated with increased concentrations of inflammatory markers^(41–43)^. The incorporation of CA, GA and 7OA in WATepi, and the higher EPA content together with the lower content of AA after intake of the HERO or the CECO diet, could be expected to affect the inflammatory cascades in adipose tissues. Although the EPA and AA concentration in WATepi were similar between HERO and CECO groups, lower WATepi concentrations of TNF*α*, MMP3, IL6, MCP1 and ITGAM were observed in CECO group but not in the HERO group when compared with the Control group. A likely explanation for this is the higher *n*-11 MUFA concentration in WATepi from the CECO group.

Inflammation and infiltration of macrophages (Kupffer cells) in liver increase with increasing accumulation of fat and the severity of the damage to the liver^(44)^. Since the obese Zucker fa/fa rat has manifested severe steatosis in liver cells at 12 weeks age^(45,46)^ and presents chronic inflammation^(25–28)^, it was of interest to investigate whether the excessive deposition of TAG in liver and the abnormal high serum alanine transaminase concentration in all groups, as well as the changes in the fatty acid composition in liver after intake of CA, can be associated with hepatic concentrations of markers of inflammation and macrophage infiltration. Neither the HERO nor the CECO diet affected liver contents of the other markers of macrophage infiltration or inflammation, that is, ITGAM, TNF*α*, MMP3, IL6 or MCP1, mainly originating from adipose tissues where the concentrations of these markers were decreased after CECO intake. The lower hepatic content of CD68, a central marker for macrophage infiltration, in the CECO group indicates that CA intake may attenuate the development of damage to the steatotic liver.

Herring oil and concentrates of CA have been tested only to a limited extent in rodents; therefore, we included a panel of safety biomarkers in the present study. The obese Zucker fa/fa rat is afflicted with metabolic disturbances that are comparable to those seen in humans^(20–22)^; therefore, it was important to investigate if these co-morbidities were affected, either adversely or beneficially, by CA intake. It seemed, however, that biomarkers for liver, heart and kidney functions were not affected by either of the CA-containing diets in the present study.

This study presents evidence that the anti-inflammatory effect of dietary CA is not mediated through increased biosynthesis of EPA in obese Zucker fa/fa rats, and the findings suggest that CA may directly affect the inflammatory cascades, especially in adipose tissues. The lower macrophage infiltration in WATepi from the CECO group, as indicated by the lower ITGAM level, may be one explanation for the lower levels of inflammatory markers in this tissue. We have recently isolated leucocytes (but not pure macrophages) from humans taking a CA supplement daily for 8 weeks, and we found quantifiable amounts of CA in the leucocyte membrane (data to be published). Accumulation of CA in the leucocyte membrane may have an impact on the characteristics of these cells, such as the activity of the cyclooxygenase system producing prostaglandins with pro- or anti-inflammatory properties. Thus, a combination of lower macrophage infiltration in WAT and altered fatty acid composition in leucocyte (and possibly macrophage) membrane may be one explanation for the observed anti-inflammatory effect of the CECO diet in the Zucker fa/fa rats in the present study. These findings are not directly transferable to humans but may have relevance as the prevalence of obesity and co-morbidities of obesity is increasing globally. We propose that the effect of consuming of CA, either from pelagic fish such as herring or from oil produced from residuals from fisheries, on inflammation and the immune system should be investigated in clinical studies.

This study has some strength and limitations. Strengths include quantification of fatty acids in a range of tissues with different characteristics and functions, and measurements of a panel of markers of inflammation and macrophage infiltration in both WAT and liver. The observed effects of herring oil on fatty acid composition and on protein concentrations of desaturases in tissues from obese non-diabetic Zucker fa/fa rats confirm our previous findings in rats with overt type 2 diabetes^(17)^. Limitations to the study include the choice of animal model used, as the effect of CA intake on markers of inflammation and macrophage infiltration may be specific to obese Zucker fa/fa rats as they present chronic inflammation^(25–28)^, and the quantification of the tissue concentrations of relevant enzymes instead of measurements of enzyme activities.

### Conclusion

In this study, we present evidence that CA may have an anti-inflammatory effect. This is based on the findings that rats fed the CECO diet had lower concentrations of five highly relevant markers of inflammation and macrophage infiltration in WATepi, and we suggest that this effect is not mediated through increased EPA biosynthesis. The HERO diet contained a similar amount of EPA but half the amount of CA compared with the CECO diet and did not affect any of the measured inflammatory markers, but had similar effect to the CECO diet on the EPA concentration in tissues.

## Supporting information

Hansen et al. supplementary material Hansen et al. supplementary material

## Supplementary material

For supplementary material/s referred to in this article, please visit https://doi.org/10.1017/S0007114525105394
