# Supplementary material for: Intake of a cetoleic acid concentrate lowered concentrations of markers of inflammation and macrophage infiltration but did probably not increase EPA biosynthesis in male obese Zucker fa/fa rats
Source: Br J Nutr. 2025 Oct 17;134(11):881–91. doi: 10.1017/S0007114525105394 (PMC12797074; doi:10.1017/S0007114525105394)
Supplement: Hansen et al. supplementary material [file S0007114525105394sup001.docx]

**Supplemental table 1**: Fatty acids in liver, presented as g/100g fatty acids (mean values and standard deviations)

|  | Control group | HERO group | CECO group | *P* ANOVA |
| --- | --- | --- | --- | --- |
| C14:0 | 1.829 ± 0.123^a^ | 1.833 ± 0.124^ab^ | 1.997 ± 0.178^b^ | 0.024 |
| C15:0 | 0.095 ± 0.013^a^ | 0.122 ± 0.017^b^ | 0.102 ± 0.013^a^ | 1.4x10^-3^ |
| C16:0 | 37.606 ± 1.333^a^ | 38.598 ± 1.862^a^ | 41.062 ± 2.034^b^ | 5.6x10^-4^ |
| C17:0 | 0.094 ± 0.010^a^ | 0.122 ± 0.008^b^ | 0.097 ± 0.01^a^ | 7.4x10^-7^ |
| C18:0 | 4.258 ± 0.673 | 4.729 ± 0.884 | 4.830 ± 0.610 | 0.19 |
| C22:0 | 0.060 ± 0.009 | 0.055 ± 0.025 | 0.043 ± 0.011 | 0.082 |
| C23:0 | 0.129 ± 0.016 | 0.137 ± 0.065 | 0.118 ± 0.027 | 0.61 |
| C14:1 n-5 | 0.181 ± 0.033^a^ | 0.132 ± 0.025^b^ | 0.135 ± 0.013^b^ | 2.6x10^-4^ |
| C16:1 n-7 | 9.019 ± 0.655^a^ | 8.016 ± 0.631^b^ | 7.437 ± 0.571^b^ | 2.2x10^-5^ |
| C17:1 n-8 | 0.134 ± 0.012^a^ | 0.158 ± 0.011^b^ | 0.109 ± 0.007^c^ | 4.8x10^-10^ |
| C16:1 n-9 | 0.689 ± 0.084^ab^ | 0.626 ± 0.071^a^ | 0.706 ± 0.048^b^ | 0.046 |
| C18:1 n-11 | <LOQ | 0.851 ± 0.052^a^ | 1.772 ± 0.204^b^ | 2.6x10^-10^ |
| C20:1 n-11 | <LOQ | 0.359 ± 0.036^a^ | 0.732 ± 0.095^b^ | 3.5x10^-09^ |
| C22:1 n-11 | <LOQ | 0.044 ± 0.029^a^ | 0.085 ± 0.034^b^ | 0.010 |
| C18:1 n-9 | 26.861 ± 1.887 | 28.064 ± 3.287 | 26.738 ± 2.556 | 0.49 |
| C20:1 n-9 | 0.087 ± 0.013^a^ | 0.215 ± 0.063^b^ | 0.340 ± 0.096^c^ | 3.4x10^-8^ |
| C22:1 n-9 | <LOQ | 0.016 ± 0.008^a^ | 0.022 ± 0.005^b^ | 0.043 |
| C18:1 n-7 | 3.707 ± 0.437^a^ | 3.059 ± 0.281^b^ | 2.669 ± 0.230^c^ | 8.1x10^-7^ |
| C18:2 n-6 | 9.573 ± 1.856^a^ | 6.919 ± 1.397^b^ | 5.975 ± 1.146^b^ | 3.9x10^-5^ |
| C18:3 n-6 | 0.273 ± 0.034^a^ | 0.140 ± 0.020^b^ | 0.141 ± 0.025^b^ | 1.0x10^-11^ |
| C20:3 n-6 | 0.245 ± 0.082 | 0.299 ± 0.187 | 0.228 ± 0.070 | 0.44 |
| C20:4 n-6 | 2.743 ± 0.550^a^ | 1.933 ± 0.910^b^ | 1.917 ± 0.598^b^ | 0.020 |
| C22:4 n-6 | 0.134 ± 0.042^a^ | 0.044 ± 0.023^b^ | 0.044 ± 0.023^b^ | 3.6x10^-7^ |
| C22:5 n-6 | 0.115 ± 0.033^a^ | 0.026 ± 0.014^b^ | 0.029 ± 0.011^b^ | 7.3x10^-10^ |
| C18:3 n-3 | 0.562 ± 0.178^a^ | 0.455 ± 0.121^ab^ | 0.362 ± 0.118^b^ | 0.016 |
| C20:5 n-3 | 0.066 ± 0.021^a^ | 0.402 ± 0.213^b^ | 0.256 ± 0.108^b^ | 5.0x10^-5^ |
| C22:5 n-3 | 0.206 ± 0.052^a^ | 0.439 ± 0.207^b^ | 0.360 ± 0.161^ab^ | 7.8x10^-3^ |
| C22:6 n-3 | 0.769 ± 0.182^a^ | 1.654 ± 0.891^b^ | 1.067 ± 0.352^ab^ | 5.6x10^-3^ |

Data are presented as mean ± standard deviation for *n* 10 in the Control group, *n* 9 in the HERO group, and *n* 10 in the CECO group.

Groups are compared using one-way ANOVA followed by Tukey HSD post hoc test when appropriate, or with Students t-test when only two groups are compared. Means in a row with different letters are significantly different (*P* < 0.05).

LOQ, level of quantification

HERO, herring oil; CECO, cetoleic acid concentrate

**Supplemental table 2**: Fatty acids in epididymal white adipose tissue, presented as g/100g fatty acids (mean values and standard deviations)

|  | Control group | HERO group | CECO group | *P* ANOVA |
| --- | --- | --- | --- | --- |
| C12:0 | 0.109 ± 0.041 | 0.139 ± 0.019 | 0.139 ± 0.025 | 0.051 |
| C14:0 | 1.635 ± 0.119^a^ | 2.264 ± 0.182^b^ | 1.880 ± 0.154^c^ | 1.0x10^-8^ |
| C15:0 | 0.082 ± 0.03^a^ | 0.132 ± 0.011^b^ | 0.099 ± 0.009^a^ | 2.3x10^-5^ |
| C16:0 | 28.328 ± 0.266^a^ | 29.303 ± 1.100^b^ | 30.027 ± 0.731^b^ | 1.6x10^-4^ |
| C17:0 | 0.117 ± 0.008^a^ | 0.137 ± 0.008^b^ | 0.115 ± 0.009^a^ | 1.4x10^-5^ |
| C18:0 | 3.634 ± 0.380 | 3.656 ± 0.633 | 3.699 ± 0.490 | 0.96 |
| C14:1 n-5 | 0.140 ± 0.030 | 0.145 ± 0.020 | 0.131 ± 0.022 | 0.46 |
| C16:1 n-7 | 7.007 ± 0.941 | 7.312 ± 1.003 | 6.737 ± 0.665 | 0.38 |
| C18:1 n-7 | 2.854 ± 0.079^a^ | 2.519 ± 0.121^b^ | 2.379 ± 0.105^c^ | 3.5x10^-10^ |
| C17:1 n-8 | 0.135 ± 0.009^a^ | 0.161 ± 0.011^b^ | 0.131 ± 0.011^a^ | 3.0x10^-6^ |
| C16:1 n-9 | 0.365 ± 0.031 | 0.377 ± 0.044 | 0.401 ± 0.023 | 0.073 |
| C18:1 n-9 | 29.834 ± 1.425^a^ | 28.935 ± 1.460^ab^ | 27.829 ± 1.355^b^ | 0.014 |
| C20:1 n-9 | 0.228 ± 0.027^a^ | 0.987 ± 0.092^b^ | 2.081 ± 0.146^c^ | 2.0x10^-24^ |
| C22:1 n-9 | <LOQ | 0.045 ± 0.007^a^ | 0.105 ± 0.015^b^ | 2.5x10^-9^ |
| C18:1 n-11 | <LOQ | 0.807 ± 0.068^a^ | 1.363 ± 0.105^b^ | 1.6x10^-10^ |
| C20:1 n-11 | <LOQ | 0.598 ± 0.089^a^ | 1.173 ± 0.126^b^ | 1.5x10^-20^ |
| C22:1 n-11 | <LOQ | 0.517 ± 0.076^a^ | 1.362 ± 0.149^b^ | 2.3x10^-11^ |
| C18:2 n-6 | 21.827 ± 1.239^a^ | 17.691 ± 0.704^b^ | 16.451 ± 0.855^c^ | 4.5x10^-12^ |
| C20:2 n-6 | 0.231 ± 0.028^a^ | 0.176 ± 0.011^b^ | 0.192 ± 0.012^b^ | 3.1x10^-6^ |
| C20:3 n-6 | 0.259 ± 0.019^a^ | 0.184 ± 0.018^b^ | 0.192 ± 0.017^b^ | 6.9x10^-10^ |
| C20:4 n-6 | 0.577 ± 0.081^a^ | 0.420 ± 0.049^b^ | 0.459 ± 0.054^b^ | 2.7x10^-5^ |
| C18:3 n-3 | 2.047 ± 0.162^a^ | 1.698 ± 0.085^b^ | 1.569 ± 0.084^b^ | 4.3x10^-9^ |
| C22:4 n-6 | 0.193 ± 0.037^a^ | 0.103 ± 0.012^b^ | 0.123 ± 0.012^b^ | 2.0x10^-8^ |
| C20:5 n-3 | 0.045 ± 0.009^a^ | 0.344 ± 0.058^b^ | 0.359 ± 0.058^b^ | 1.3x10^-14^ |
| C22:5 n-3 | 0.181 ± 0.027^a^ | 0.477 ± 0.059^b^ | 0.512 ± 0.072^b^ | 3.7x10^-13^ |
| C22:6 n-3 | 0.153 ± 0.038^a^ | 0.874 ± 0.137^b^ | 0.493 ± 0.061^c^ | 2.1x10^-15^ |

Data are presented as mean ± standard deviation for *n* 10 in the Control group, *n* 9 in the HERO group, and *n* 10 in the CECO group.

Groups are compared using one-way ANOVA followed by Tukey HSD post hoc test when appropriate, or with Students t-test when only two groups are compared. Means in a row with different letters are significantly different (*P* < 0.05).

LOQ, level of quantification

HERO, herring oil; CECO, cetoleic acid concentrate

**Supplemental table 3**: Fatty acids in skeletal muscle, presented as g/100g fatty acids (mean values and standard deviations)

|  | Control group | HERO group | CECO group | *P* ANOVA |
| --- | --- | --- | --- | --- |
| C12:0 | 0.114 ± 0.036 | 0.104 ± 0.031 | 0.118 ± 0.045 | 0.72 |
| C14:0 | 1.266 ± 0.455 | 1.644 ± 0.459 | 1.506 ± 0.377 | 0.17 |
| C15:0 | 0.077 ± 0.013^a^ | 0.109 ± 0.018^b^ | 0.096 ± 0.037^ab^ | 0.029 |
| C16:0 | 26.722 ± 2.075^a^ | 27.306 ± 2.728^ab^ | 30.162 ± 3.926^b^ | 0.039 |
| C17:0 | 0.166 ± 0.053 | 0.175 ± 0.032 | 0.170 ± 0.046 | 0.90 |
| C18:0 | 9.217 ± 3.487 | 8.147 ± 2.495 | 8.305 ± 2.527 | 0.68 |
| C14:1 n-5 | 0.137 ± 0.087 | 0.144 ± 0.068 | 0.143 ± 0.064 | 0.98 |
| C16:1 n-7 | 5.747 ± 3.246 | 6.081 ± 2.427 | 5.769 ± 2.618 | 0.96 |
| C18:1 n-7 | 3.018 ± 0.193^a^ | 2.751 ± 0.182^b^ | 2.642 ± 0.179^b^ | 3.4x10^-4^ |
| C18:1 n-9 | 16.075 ± 5.435 | 17.016 ± 5.113 | 14.941 ± 5.093 | 0.69 |
| C20:1 n-9 | 0.146 ± 0.063^a^ | 0.600 ± 0.145^b^ | 0.941 ± 0.288^c^ | 4.9x10^-9^ |
| C22:1 n-9 | 0.37 ± 0.209 | 0.392 ± 0.243 | 0.288 ± 0.089 | 0.46 |
| C18:1 n-11 | <LOQ | 0.431 ± 0.073^a^ | 0.732 ± 0.281^b^ | 6.4x10^-3^ |
| C20:1 n-11 | <LOQ | 0.321 ± 0.101^a^ | 0.528 ± 0.247^b^ | 3.2x10^-2^ |
| C22:1 n-11 | <LOQ | 0.446 ± 0.178^a^ | 0.650 ± 0.204^b^ | 3.410^-2^ |
| C18:2 n-6 | 19.019 ± 1.739^a^ | 16.88 ± 2.521^ab^ | 15.119 ± 1.886^b^ | 1.1x10^-3^ |
| C20:3 n-6 | 0.618 ± 0.200 | 0.502 ± 0.153 | 0.538 ± 0.175 | 0.36 |
| C20:4 n-6 | 8.083 ± 3.437^a^ | 4.906 ± 1.994^b^ | 5.754 ± 2.025^ab^ | 0.033 |
| C22:4 n-6 | 0.672 ± 0.168^a^ | 0.169 ± 0.042^b^ | 0.244 ± 0.048^b^ | 7.9x10^-11^ |
| C18:3 n-3 | 0.965 ± 0.519 | 0.752 ± 0.284 | 0.614 ± 0.341 | 0.16 |
| C20:5 n-3 | 0.126 ± 0.045^a^ | 0.556 ± 0.136^b^ | 0.588 ± 0.152^b^ | 3.0x10^-9^ |
| C22:5 n-3 | 2.386 ± 0.994^ab^ | 2.046 ± 0.593^a^ | 3.135 ± 1.113^b^ | 0.049 |
| C22:6 n-3 | 5.049 ± 2.314^a^ | 8.523 ± 3.023^b^ | 7.017 ± 2.809^ab^ | 0.033 |

Data are presented as mean ± standard deviation for *n* 10 in the Control group, *n* 9 in the HERO group, and *n* 10 in the CECO group.

Groups are compared using one-way ANOVA followed by Tukey HSD post hoc test when appropriate, or with Students t-test when only two groups are compared. Means in a row with different letters are significantly different (*P* < 0.05).

LOQ, level of quantification

HERO, herring oil; CECO, cetoleic acid concentrate

**Supplemental table 4**: Fatty acids in blood cells, presented as g/100g fatty acids (mean values and standard deviations)

|  | Control group | HERO group | CECO group | *P* ANOVA |
| --- | --- | --- | --- | --- |
| C14:0 | 0.944 ± 0.215 | 1.222 ± 0.443 | 1.141 ± 0.273 | 0.17 |
| C15:0 | 0.124 ± 0.011^a^ | 0.161 ± 0.021^b^ | 0.136 ± 0.015^a^ | 1.0x10^-4^ |
| C16:0 | 20.937 ± 1.353^a^ | 21.721 ± 0.97^ab^ | 22.171 ± 0.413^b^ | 0.031 |
| C17:0 | 0.088 ± 0.007^a^ | 0.098 ± 0.014^a^ | 0.077 ± 0.007^b^ | 2.7x10^-4^ |
| C18:0 | 4.956 ± 0.876 | 5.202 ± 0.962 | 4.924 ± 0.649 | 0.74 |
| C24:0 | 0.696 ± 0.336 | 0.654 ± 0.211 | 0.466 ± 0.133 | 0.10 |
| C14:1 n-5 | 0.067 ± 0.034 | 0.075 ± 0.050 | 0.070 ± 0.030 | 0.90 |
| C16:1 n-7 | 3.366 ± 0.945 | 3.768 ± 1.235 | 3.438 ± 0.654 | 0.63 |
| C18:1 n-7 | 1.436 ± 0.083^a^ | 1.382 ± 0.121^ab^ | 1.292 ± 0.101^b^ | 0.014 |
| C16:1 n-9 | 0.346 ± 0.093 | 0.353 ± 0.062 | 0.392 ± 0.064 | 0.35 |
| C18:1 n-9 | 5.479 ± 0.801^a^ | 6.433 ± 0.718^ab^ | 6.212 ± 0.882^b^ | 0.038 |
| C20:1 n-9 | 0.029 ± 0.006^a^ | 0.097 ± 0.034^b^ | 0.227 ± 0.072^c^ | 2.6x10^-9^ |
| C22:1 n-9 | <LOQ | 0.015 ± 0.004^a^ | 0.020 ± 0.008^b^ | 1.910^-3^ |
| C24:1 n-9 | 0.281 ± 0.127^a^ | 0.546 ± 0.229^b^ | 0.552 ± 0.205^b^ | 5.3x10^-3^ |
| C18:1 n-11 | <LOQ | 0.094 ± 0.040^a^ | 0.194 ± 0.059^b^ | 5.0x10^-4^ |
| C20:1 n-11 | <LOQ | 0.015 ± 0.014^a^ | 0.052 ± 0.028^b^ | 2.3x10^-3^ |
| C22:1 n-11 | <LOQ | 0.063 ± 0.042^a^ | 0.194 ± 0.099^b^ | 1.9x10^-3^ |
| C18:2 n-6 | 14.101 ± 0.956 | 13.784 ± 1.280 | 13.225 ± 0.648 | 0.15 |
| C18:3 n-6 | 0.392 ± 0.145 | 0.307 ± 0.077 | 0.301 ± 0.022 | 0.083 |
| C20:3 n-6 | 0.922 ± 0.180 | 1.033 ± 0.083 | 0.968 ± 0.129 | 0.23 |
| C20:4 n-6 | 37.627 ± 2.518^a^ | 27.810 ± 4.420^b^ | 29.915 ± 2.291^b^ | 6.6x10^-7^ |
| C22:4 n-6 | 0.843 ± 0.065^a^ | 0.464 ± 0.043^b^ | 0.509 ± 0.023^b^ | 3.1x10^-16^ |
| C22:5 n-6 | 0.679 ± 0.103^a^ | 0.305 ± 0.027^b^ | 0.346 ± 0.045^b^ | 2.4x10^-12^ |
| C18:3 n-3 | 0.602 ± 0.217 | 0.568 ± 0.353 | 0.507 ± 0.149 | 0.70 |
| C20:5 n-3 | 0.858 ± 0.191^a^ | 4.862 ± 0.592^b^ | 4.921 ± 0.377^b^ | 6.2x10^-19^ |
| C22:5 n-3 | 1.586 ± 0.120^a^ | 2.078 ± 0.132^b^ | 2.429 ± 0.178^c^ | 4.2x10^-12^ |
| C22:6 n-3 | 3.590 ± 0.302^a^ | 6.850 ± 0.516^b^ | 5.275 ± 0.307^c^ | 9.6x10^-16^ |

Data are presented as mean ± standard deviation for *n* 10 in the Control group, *n* 9 in the HERO group, and *n* 10 in the CECO group.

Groups are compared using one-way ANOVA followed by Tukey HSD post hoc test when appropriate, or with Students t-test when only two groups are compared. Means in a row with different letters are significantly different (*P* < 0.05).

LOQ, level of quantification

HERO, herring oil; CECO, cetoleic acid concentrate

**Supplemental table 5**: Fatty acids in brain, presented as g/100g fatty acids (mean values and standard deviations)

|  | Control group | HERO group | CECO group | *P* ANOVA |
| --- | --- | --- | --- | --- |
| C14:0 | 0.173 ± 0.017 | 0.183 ± 0.025 | 0.177 ± 0.022 | 0.60 |
| C16:0 | 19.568 ± 3.479 | 20.873 ± 2.004 | 19.664 ± 2.854 | 0.56 |
| C17:0 | 0.131 ± 0.013 | 0.141 ± 0.013 | 0.141 ± 0.013 | 0.19 |
| C18:0 | 22.603 ± 2.648 | 23.647 ± 2.010 | 22.110 ± 2.396 | 0.37 |
| C20:0 | 1.070 ± 0.638 | 0.787 ± 0.439 | 0.963 ± 0.525 | 0.53 |
| C22:0 | 1.246 ± 0.840 | 0.842 ± 0.519 | 1.083 ± 0.642 | 0.45 |
| C23:0 | 0.896 ± 0.132^a^ | 0.749 ± 0.114^b^ | 0.779 ± 0.105^ab^ | 0.026 |
| C24:0 | 2.287 ± 1.640 | 1.525 ± 0.985 | 2.099 ± 1.268 | 0.45 |
| C16:1 n-7 | 0.517 ± 0.080 | 0.546 ± 0.065 | 0.528 ± 0.109 | 0.77 |
| C18:1 n-7 | 3.309 ± 0.906 | 2.904 ± 0.484 | 3.426 ± 0.811 | 0.32 |
| C20:1 n-7 | 0.544 ± 0.490 | 0.345 ± 0.306 | 0.542 ± 0.421 | 0.50 |
| C22:1 n-7 | 0.196 ± 0.200 | 0.102 ± 0.111 | 0.169 ± 0.159 | 0.44 |
| C16:1 n-9 | 0.125 ± 0.032 | 0.131 ± 0.020 | 0.123 ± 0.032 | 0.83 |
| C18:1 n-9 | 13.837 ± 2.440 | 13.218 ± 1.642 | 14.590 ± 2.377 | 0.41 |
| C20:1 n-9 | 1.590 ± 1.409 | 1.072 ± 0.997 | 1.651 ± 1.324 | 0.56 |
| C24:1 n-9 | 2.680 ± 2.486 | 1.619 ± 1.422 | 2.579 ± 2.044 | 0.48 |
| C22:1 n-9 | 0.287 ± 0.220 | 0.185 ± 0.143 | 0.264 ± 0.183 | 0.47 |
| C18:2 n-6 | 0.942 ± 0.178 | 0.877 ± 0.104 | 0.904 ± 0.218 | 0.72 |
| C20:3 n-6 | 0.399 ± 0.123 | 0.426 ± 0.033 | 0.451 ± 0.089 | 0.45 |
| C20:4 n-6 | 10.197 ± 2.644 | 10.378 ± 1.497 | 9.442 ± 1.999 | 0.59 |
| C22:4 n-6 | 3.423 ± 0.480^a^ | 3.111 ± 0.337^ab^ | 2.936 ± 0.403^b^ | 0.043 |
| C22:5 n-6 | 1.136 ± 0.491^a^ | 0.832 ± 0.222^ab^ | 0.710 ± 0.314^b^ | 0.041 |
| C20:5 n-3 | 0.010 ± 0.009^a^ | 0.048 ± 0.012^b^ | 0.029 ± 0.015^c^ | 2.3x10^-6^ |
| C22:5 n-3 | 0.584 ± 0.082^a^ | 0.685 ± 0.086^b^ | 0.687 ± 0.071^b^ | 0.011 |
| C22:6 n-3 | 12.249 ± 2.292 | 14.774 ± 1.872 | 13.950 ± 2.651 | 0.068 |

Data are presented as mean ± standard deviation for *n* 10 in the Control group, *n* 9 in the HERO group, and *n* 10 in the CECO group.

Groups are compared using one-way ANOVA followed by Tukey HSD post hoc test when appropriate. Means in a row with different letters are significantly different (*P* < 0.05).

HERO, herring oil; CECO, cetoleic acid concentrate
